# Vienna Letter

**Published:** 1890-03

**Authors:** Hugh Hagan

**Affiliations:** Vienna, Austria


### VIENNA LETTER.

*To the Southern Medical Journal.*

VIENNA, AUSTRIA, January 25, 1890.

La Grippe, or the Russian Influenza, since I wrote to you last, has assummed a severer form and character. The general symptoms being aggrevated, and the complication more frequent. The lobar, lobular pneumonias, bronchitis and capillary bronchitis have been the most frequent complications.

In my daily visits to the dead house, I have had much explained. Many cases that had been diagnosed Russian Influenza, were found to be something else. I have questioned the doctors and pathologists in regard to the newspaper reports of the great mortality attending influenza, and I have found but few authentic cases reported, where death could be laid at the feet of influenza. The few fatal cases that were the results of influenza, were either the very old or very young. Here we all know, is a vulnerable point for most any disease.

The number of cases reported, are now rapidly decreasing, and if the weather keeps fine and the sun remains shining, Vienna will soon be herself again.

Cocaine, as a successful substitute for ether, and chloroform exemplified, in many operations for laryngotomy and trachœtomy.

Professor Schrotter, the head of one of the largest throat clinics in Europe, has for a long time used the hydrochlorate of cocaine injections as an anæsthetic in his operations on the larynx and trachea. For his injections he uses the ordinary 10 per cent. solution and injects in the line of the incision, from one or more points. He has operated many hundred times and without exception, finds cocaine solution of this strength to be pefectly satisfactry. He operates on old and young alike, only the extreme youth being exempt, here he generally gives a few inhalations of chloroform. I have seen him perform 10 tracheotomies on patients varying from 12 years to 60, and I must ray, no anæsthetic could have given more complete satisfation. By the use of cocaine one wards off the danger of pneumonias, heart paralysis, pneumogastic paralysis, and last but not least, the discomforts following the administration of the vapors. There are many other advantages in favor of cocaine in these two operations.

Firstly the patient can be prepared in two minutes for the operation.

Secondly, in two minutes after the operation, the patient is sitting up perfectly himself. There is no nausea vomiting, suspended resperation, falling back of tongue and the hundred of accidents that can accompany the use of the ordinary anaesthetics. The patients bear with great composure the operation, and do not sustain that severe nervous strain attendant upon the administration of ether or chloroform.

Professor Schrotter is profuse in his praises of its efficacy, and I can say that, judging from its effect in the ten cases I saw, that to assume the risks of the vaporous anaesthetics were idle.

I cannot resist the temptation to write you the ways and means used here of meeting different pathological conditions, and to do this I have to take my readers to each special ward. Now, for instance, after the lecture last Thursday morning, Professor Kaposi took his class to the continuous bath room. Now we read of the continuous bath in our many authorities, but few of us have ever seen the administration or the mechanism of the bed and tub, or of its especial application. The apparatus called a tub, or bath, consists of a copper or lead lined chest, about three and a half feet wide, seven feet long and four feet deep. At the head and foot of this tub are a wheel and axle, with the cog. Four chains are attached, one to each end of the axle, and to the free end of these chains is attached a wire bed, just large enough to leave about an inch on either side. On this wire bed, or better, spring bed, a rubber air bed is laid and made fast.

The patient is laid on the bed, and can be lowered to any depth that may be desired. The water is kept at an even temperature, and often changed, the temperature to be decided by the temperature of the patient and his feelings. The patient is of course naked, and to protect him from exposure there are sectioned lids, that can cover the chest to any extent desired. Generally, only the head, upper chest and arms are not covered.

The uses of this bath are varied. Extensive burns of second or third degree, or even worse, if the patient be alive, the bath is the best known means of giving the unfortunate comfort. For at once the bath serves a dual part, that of excluding air, and an easy and evenly distributed pressure. The bath is equally good in cases of decrepitude, high temperatures, etc.

The hospital staff think so well of this mode of treatment that there are about ten of these tubs in this room, and generally they are all filled. A watch is ever present to prevent drowning.

The pathologists were a little puzzled over a case that Professor Kaposi had for section a few days ago. The patient, a male 40 years old, very thin and anaemic. His face, hands, forearms, feet and legs were natural, there being no sign of disease. The whole trunk, upper arm to elbow, and thighs to knee, was melanotic, the pigmentation being a black or brown. This whole pigmented area was a mass of cicatrices, scabs, pustules, abscesses, and hard, shot-like tubercles. Indeed, so strange a picture did the subject present, that of the 50,000 sections that Professor Kundrath had seen, never had he seen such a case. Professor Kaposi, a charming, humorous little man, began quizzing the pathologists as to its possible classification, and without exception they said they could not give a certain diagnosis. Some suggested syphilis, and some this, and others that, when at last the Professor gave them the history, as follows: The patient, a man 40 years old, formerly a very healthy but dissipated man, acquired the opium habit. He took from 10 to 15 grains of morphine daily, subcutaneously, this making from 20 to 30 punctures with the needle.

His health began to fail, with loss of flesh, when he noticed at the point of injection a small red nodule would form.
This would develop into a pustule, rupture, scab, and leave a scar which, in its turn, would become darkly pigmented, and the pigment would spread. The diagnosis was a melanosis, resulting from the effects of the irritation produced by the needle and morphine on a system so depraved. The case, so badly portrayed, was classed as a dermatological curiosity.

Before I close I must give you a few of Professor Nothnagel's points on the treatment of acute articular rheumatism, He is most heroic, and I know some of my readers will almost doubt the sanity of such heroism.

The main points in the treatment of the acute articular rheumatism, whether mean, poly, or panarthritic, are three, viz.: the enormous amount of salicylic acid, salicylate of soda, and salol he uses. Of salicilic acid, which he places ahead of all drugs in efficiency, he gives an ordinary adult, whether male or female, one gramme, or 15.5 grains every 2 hours through the 24, thus the patient takes daily 12 grammes, or 186 grains daily. He administers the drug in capsules, followed by a large glass of fluid, be it water, milk, or whatever the conditions may call. The patient continues to take this as long as the disease remains. The contra indications for such medicamen taken are infancy, fatty or weak heart, and acute gastretis. To the young he doses proportionataly to the age. The possible ostitis is so far remote that he hardly considers it.

If he decides to give the salicylate of soda, he does in the same doses, that is, 12 grammes daily, and under the same conditions and restrictions.

Now for a suggestion. Of salol, a sister drug to the above mentioned two, he gives, in the enormous doses of 1-2 gramme, or of 7 1-2 grain every two hours, under the same conditions. This last named drug often gives most grateful results.

These points, I think, will prove profitable to whomsoever may see fit to try them, for they are based upon a very large and varied experience. Professor Nothnagel is one of the best men in all Europe, his clinic most extensive. The Professor thinks the fraternity, in general, do not give the drugs to their full physiological effects, and therefore do not value them enough. He also spoke of the iodide of potash, the alkaline treatment, baths, and to the end of the chapter, but gave the first place to the three mentioned drugs. He thinks they have a more decided effect in warding off a chronic form, and thus lessening the possible ankylosis. He did not seem to think that any drug had the power to prevent such complications as myocarditis, endocarditis, pericardetis and plenutis, but that if any had such powers, these drugs should be tried before others.

Of the treatment of the complications, he did not differ materially from the general medical practice, only he spoke very emphatically over the value of the ice bag in pericarditis. The ice bag is simply a soft India rubber bag, with an adjustable plug, or clasp, that can be made fast. The bag is filled with cracked ice, and placed over the precardial region, with the thickness of an ordinary toilet towel between the chest wall and bag. This is to be kept in place until the rales disappear. Of course if there be much effusion into the percardial sack, vesicants are in place.

In wandering around, I can learn nothing new that is very recently discovered, and have failed to find any especial activity in the medical world. HUGH HAGAN, M. D.

### SALOL IN GONORRHŒA.

Dr. Dreyfous has treated several cases of gonorrhœa with salol. The doses varied from five, seven to eight grams. The discharge was less abundant. In a recent case, in which gonorrhœa appeared some time before seeking advice, Dr. Dreyfous obtained a complete cure in a few days. M. Dreyfous has tested the effect of salol administered alone, and in other instances he has given simultaneously with copaiba and cubebs in order to hasten the cure. He recommends the use of salol to surgeons who operate on the urinary organs; it renders urine aseptic, which is thus innocuous when in contact with raw surfaces. Aseptic and antiseptic conditions result from giving salol internally.—*Brit. Med. Journal.*

